# Preoperative Albuminuria and Intraoperative Chloride Load: Predictors of Acute Kidney Injury Following Major Abdominal Surgery

**DOI:** 10.3390/jcm7110431

**Published:** 2018-11-09

**Authors:** Diamantina Marouli, Kostas Stylianou, Eleftherios Papadakis, Nikolaos Kroustalakis, Stavroula Kolyvaki, Georgios Papadopoulos, Christos Ioannou, Alexandra Papaioannou, Eugene Daphnis, Dimitris Georgopoulos, Helen Askitopoulou

**Affiliations:** 1 Departments of Anaesthesiology, University Hospital of Heraklion, 71110 Crete, Greece; marouli_matina@yahoo.gr (D.M.); elepapadakis@gmail.com (E.P.); alpapa@med.uoc.gr (A.P.); askitop@gmail.com (H.A.); 2 Intensive Care, University Hospital of Heraklion, 71110 Crete, Greece; georgop@med.uoc.gr; 3 Nephrology, University Hospital of Heraklion, 71110 Crete, Greece; nkroustalakis@yahoo.gr (N.K.); eugenedaphnis@yahoo.gr (E.D.); 4 Clinical Biochemistry, University Hospital of Heraklion, 71110 Crete, Greece; s.kolyvaki@gmail.com; 5 Vascular Surgery, University Hospital of Heraklion, 71110 Crete, Greece; gpamd2003@yahoo.gr (G.P.); ioannou@med.uoc.gr (C.I.)

**Keywords:** albuminuria, chloride, postoperative acute kidney injury

## Abstract

Background: Postoperative Acute Kidney Injury (AKI) is a common and serious complication associated with significant morbidity and mortality. While several pre- and intra-operative risk factors for AKI have been recognized in cardiac surgery patients, relatively few data are available regarding the incidence and risk factors for perioperative AKI in other surgical operations. The aim of the present study was to determine the risk factors for perioperative AKI in patients undergoing major abdominal surgery. Methods: This was a prospective, observational study of patients undergoing major abdominal surgery in a tertiary care center. Postoperative AKI was diagnosed according to the Acute Kidney Injury Network criteria within 48 h after surgery. Patients with chronic kidney disease stage IV or V were excluded. Logistic regression analysis was used to evaluate the association between perioperative factors and the risk of developing postoperative AKI. Results: Eleven out of 61 patients developed postoperative AKI. Four intra-operative variables were identified as predictors of AKI: intra-operative blood loss (*p* = 0.002), transfusion of fresh frozen plasma (*p* = 0.004) and red blood cells (*p* = 0.038), as well as high chloride load (*p* = 0.033, cut-off value > 500 mEq). Multivariate analysis demonstrated an independent association between AKI development and preoperative albuminuria, defined as a urinary Albumin to Creatinine ratio ≥ 30 mg·g^−1^ (OR = 6.88, 95% CI: 1.43–33.04, *p* = 0.016) as well as perioperative chloride load > 500 mEq (OR = 6.87, 95% CI: 1.46–32.4, *p* = 0.015). Conclusion: Preoperative albuminuria, as well as a high intraoperative chloride load, were identified as predictors of postoperative AKI in patients undergoing major abdominal surgery.

## 1. Introduction

Postoperative acute kidney injury (AKI) represents a common, yet under-recognized, perioperative complication, with a reported incidence varying between 1% and 36%, depending on the type of surgery and the definition of kidney failure [1,2,3,4]. AKI represents a leading cause of morbidity and mortality in surgical patients [5,6,7], and an increased risk for chronic kidney disease (CKD) and hemodialysis after discharge, factors associated with increased cost and resource utilization [8]. Even minor postoperative creatinine increases not fulfilling AKI criteria are independently associated with a two-fold risk of in-hospital death and a three-day longer hospital stay [9]. Although risk factors for postoperative AKI in patients undergoing cardiac surgery have been extensively studied [10,11], there is a relative paucity of available data regarding perioperative risk factors associated with major non-cardiac surgery.

The aim of this prospective observational study was to determine the incidence and to identify the pre- and intra-operative risk factors associated with the development of postoperative AKI, using the Acute Kidney Injury Network (AKIN) criteria [12], in patients undergoing elective major abdominal surgery.

## 2. Methods

### 2.1. Study Population

This was a single-center, prospective cohort study conducted at a tertiary academic hospital. Between February 2012 and December 2016 all adult patients undergoing elective major abdominal (including vascular) surgery were enrolled. All subjects gave their informed consent for inclusion before they participated in the study. The study was conducted in accordance with the Declaration of Helsinki, and the protocol was approved by the local institutional Ethics Committee (Approval-Nr. 5985, 14.07.2011)

On admission to hospital, the following patient variables were recorded: Age, sex, body mass index, preoperative medications, American Society of Anesthesiologists (ASA) physical status [13], and co-morbidities (arterial hypertension, coronary artery disease, congestive heart failure, chronic obstructive pulmonary disease, and diabetes mellitus). Perioperative evaluation of renal function included measurements of the following laboratory parameters: Serum creatinine and cystatin C, as well as urine albumin and creatinine at predefined time points. The times of measurements were: Preoperatively (Pre-op), in the recovery room (RR), and on postoperative days (POD) 1, 3, 5 and 7 (POD1, POD3, POD5, POD7). The calculated parameters were: Estimated glomerular filtration rate (eGFR), urine albumin to creatinine ratio (UACR), fractional excretion of sodium (FeNa), and fractional excretion of urea (FeUr). An eGFR < 90 mL·min·m^−2^ and a UACR value ≥ 30 mg·g^−1^ were considered abnormal. Serum and urine samples were centrifuged at 3000 rpm for 10 min and at 1500 rpm for 5 min, respectively, and stored at −80 °C until analysis. Exclusion criteria included: ASA 5 (moribund, not expected to live 24 h irrespective of procedure), chronic kidney disease (CKD) stage IV or V [14], preoperative (within the prior month) use of drugs with known nephrotoxic activity, and emergency surgery.

The recorded intra-operative surgical and anesthetic parameters were: Type of surgery, volume and type of intravenous fluids, transfusions of red blood cells, fresh frozen plasma or platelets, vasopressor administration, mean arterial blood pressure, urine output, and blood loss. The total chloride ion content of intra-operatively administered crystalloids and colloids was estimated. Patients were followed until the 7th postoperative day or hospital discharge, whatever occurred first. Preoperative patient demographics, laboratory values and intra-operative variables were evaluated for their association with perioperative AKI development.

### 2.2. Outcomes

The primary outcome of the study was the development of perioperative AKI, defined by the AKIN criteria using the maximal change in serum creatinine and eGFR within 48 h after surgery (Table 1). The eGFR was estimated by using the Chronic Kidney Disease Epidemiology (CKD-EPI) Creatinine Equation [15].

### 2.3. Statistical Analysis

Data were analyzed using SPSS for Windows (version 22.0; SPSS Inc., Chicago, IL, USA). Continuous variables were expressed as medians with range or means ± SD and were analyzed using an independent *t*-test or the Mann-Whitney test. Numerical data, expressed as counts and % proportions, were analyzed using the Pearson’s chi-square or the Fisher’s exact test. Simple logistic regression models were applied to determine odds ratios (ORs) and 95% confidence intervals (CIs), for the perioperative risk factors associated with postoperative AKI development. Multiple logistic regression was applied to establish a model for AKI prediction with prediction variables UACR > 30 mg·g^−1^ and intraoperative chloride load > 500 mEq.

For graphical representation, data box and whisker plots were used. For all analyses, a *p* value < 0.05 was considered statistically significant.

## 3. Results

Between February 2012 and December 2016, 61 patients (47 males and 14 females) with a mean age of 67.1 ± 10.2 years underwent major elective abdominal surgery. Eleven patients (18%) developed postoperative AKI (AKI group), while 50 did not (Non-AKI group). The baseline clinical and demographic characteristics of both groups are presented in Table 2, while preoperative laboratory values and intraoperative variables are presented in Table 3 and Table 4, respectively. The two groups were comparable in terms of sex, age, body mass index, co-morbidities, major drug categories prescription, pre-operative CKD stage, and ASA score. However, a significant association between type of surgery and development of perioperative AKI was detected, with vascular surgery patients experiencing a significantly higher AKI incidence (31.8% vs. 10.3% in non-vascular surgery patients).

**Figure 1:**
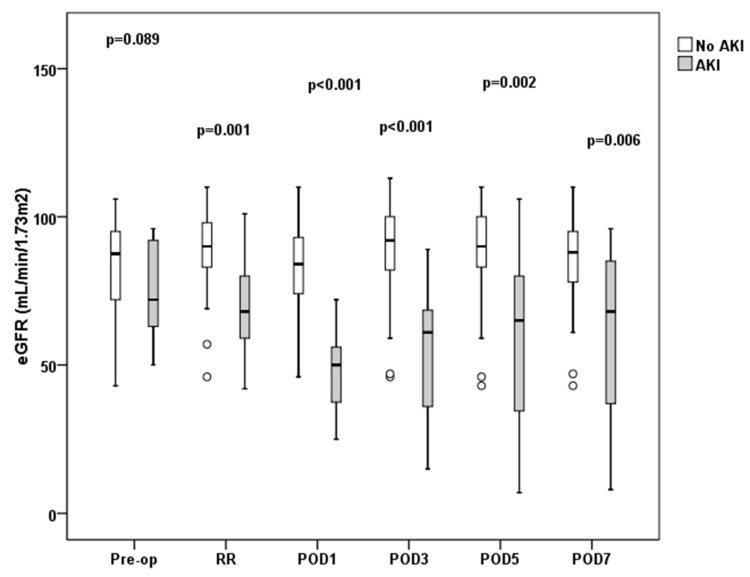
Estimated glomerular filtration rate (eGFR) at predefined time-points. Open boxes represent non-AKI patients, shaded boxes AKI patients. Boxes and whiskers show interquartile ranges and total observed ranges, respectively. eGFR: estimated Glomerular Filtration Rate; Pre-op: preoperatively; RR: recovery room; POD1: postoperative day 1; POD3: postoperative day 3; POD5: postoperative day 5; POD7: postoperative day 7.

There was no statistically significant difference in the preoperative eGFR between AKI and non-AKI patients (75.3 ± 16 vs. 83.9 ± 15.2 mL·min^−1^·m^−2^). However, in the AKI group, the eGFR was significantly lower in the recovery room (69.5 ± 18.7 vs. 85.7 ± 15.6 mL·min^−1^·m^−2^, *p* = 0.001) and remained so throughout the entire follow-up period (Figure 1).

**Figure 2:**
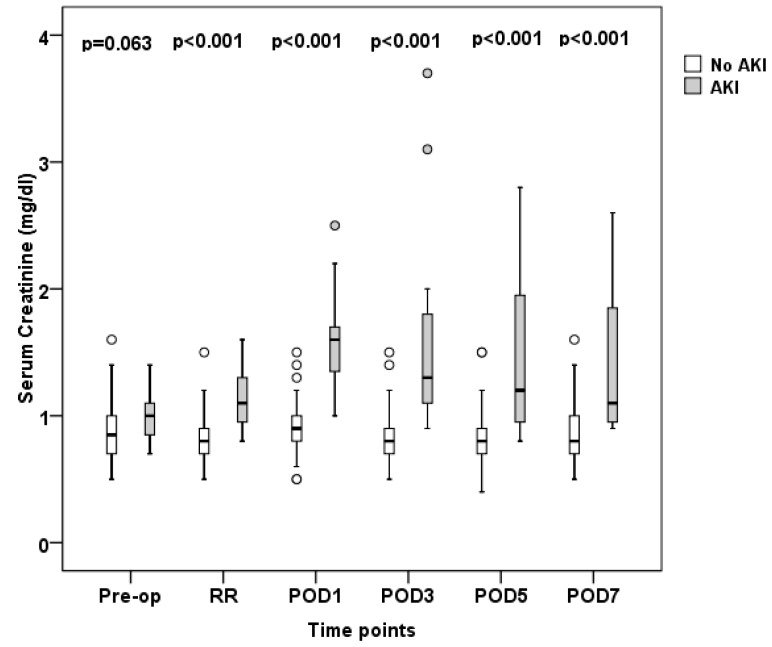
Serum creatinine measurements at predefined time-points. Open boxes represent non-AKI patients, shaded boxes AKI patients. Boxes and whiskers show interquartile ranges and total observed ranges, respectively. Pre-op: preoperatively; RR: recovery room; POD1: postoperative day 1; POD3: postoperative day 3; POD5: postoperative day 5; POD7: postoperative day 7.

**Figure 3:**
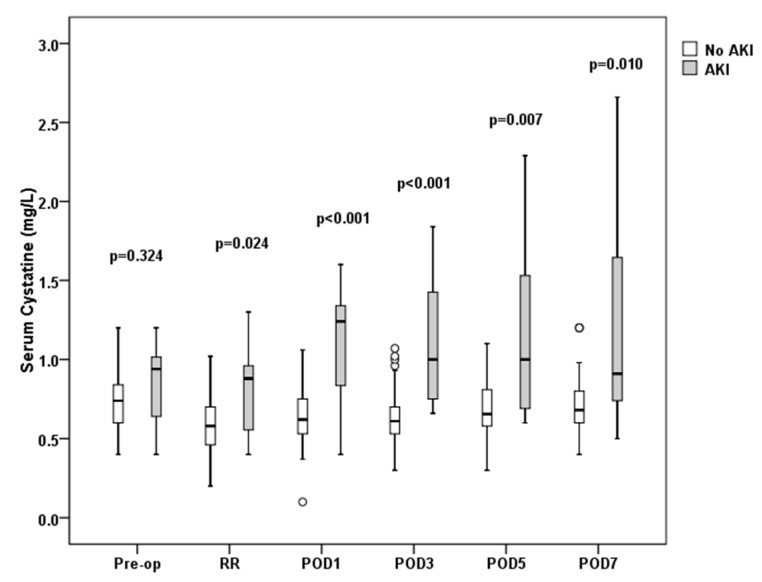
Serum cystatin C measurements at predefined time-points. Open boxes represent non-AKI patients, shaded boxes AKI patients. Boxes and whiskers show interquartile ranges and total observed ranges, respectively. Pre-op: preoperatively; RR: recovery room; POD1: postoperative day 1; POD3: postoperative day 3; POD5: postoperative day 5; POD7: postoperative day 7.

Differences between AKI and non-AKI patients regarding serum creatinine and cystatin C concentrations are shown in Figure 2 and Figure 3, respectively. 

Univariate analysis showed that an abnormal *preoperative* UACR was strongly associated with postoperative AKI development (OR = 5.47, 95% CI: 1.36–21.92, *p* = 0.019). Four *intra-operative* variables were also identified as significant predictors of postoperative AKI (Table 4): intra-operative blood loss, transfusion of RBCs and FFP, and intraoperative chloride load. Receiver operating curve analysis showed that an intraoperative chloride load > 500 mEq (AUC 0.715 ± 0.095, *p* = 0.033) had a sensitivity of 70% and a specificity of 77% in predicting the development of AKI. There was a strong correlation between blood losses and transfusion of RBCs and FFP. Thus, due to co-linearity, only the total amount of intraoperative fluids, along with chloride load and UACR were introduced in the multivariate model. Multivariate regression analysis confirmed that a UACR ≥ 30 mg·g^−1^ (OR = 6.88, 95% CI: 1.43–33.04, *p* = 0.016) and chloride load > 500 mEq (OR = 6.87, 95% CI: 1.46–32.4, *p* = 0.015) were independent predictors of postoperative AKI. The remaining intra-operative variables did not differ significantly between the two groups.

## 4. Discussion

The present prospective study of postoperative acute kidney injury following elective major abdominal surgery showed an incidence of 18%. The development of AKI, defined by the AKIN creatinine criteria, was strongly associated with an abnormal (i.e., ≥30 mg·g^−1^) preoperative urine albumin to creatinine ratio. Furthermore, four intra-operative variables were identified as independent AKI predictors: Intra-operative blood loss, transfusion of red blood cells and fresh frozen plasma, and intraoperative chloride load >500 mEq. 

The incidence of postoperative AKI and its association with perioperative risk factors has been extensively studied in the cardiac surgery population [16]. In the majority of these studies, postoperative AKI was defined as deterioration in renal function requiring renal replacement therapy in patients without pre-existing kidney dysfunction. However, risk factors for AKI following major abdominal surgery have received less attention. According to a recent systematic review of risk prediction models for AKI following non-cardiac surgery, seven models were identified from six studies [17], with only two studies focusing on general surgery patients. Kheterpal et al. [18] studied the incidence and risk factors for postoperative AKI in a general population of non-cardiac (mainly thoracic, intraperitoneal, and suprainguinal vascular) surgery patients with normal renal function (defined as a calculated preoperative creatinine clearance of ≥80 mL·min^−1^). They found an AKI incidence of 0.8% and identified seven independent preoperative AKI risk factors: Age, body mass index, liver disease, peripheral vascular disease, chronic obstructive pulmonary disease, high-risk surgery, and emergent surgery. The low incidence of postoperative AKI in that study was probably related to the definition of AKI as a drop in estimated creatinine clearance below 50 mL·min^−1^, within the first 7 postoperative days. Biteker et al. [19] studied postoperative AKI, as defined by the RIFLE [20] AKI staging criteria in elective non-cardiac major surgery patients without preoperative renal dysfunction (serum creatinine <1.6 mg·dL^−1^ for men and <1.4 mg·dL^−1^ for women, respectively). They found a postoperative AKI incidence of 6.7% and identified four independent AKI predictors (age, diabetes, Revised Cardiac Risk Index [21], and ASA physical status).

In the present study, the incidence of AKI was 18%, significantly higher compared to the aforementioned studies. This could be partly explained by the different definitions used for the diagnosis of AKI. It is well known that the AKIN classification (by “broadening” the stage I AKI criteria) increases the sensitivity of AKI diagnosis. In fact, Joannidis et al. [22] showed that by using the AKIN classification, an additional 9% of cases not fulfilling RIFLE criteria could be identified. Furthermore, the present study excluded only patients with stage IV and V chronic kidney disease and patients receiving drugs with established nephrotoxic effects [23]. In fact, 49.2% and 11.5% of the studied population had CKD stage II and III, respectively. This factor should be considered when interpreting our results, since a well-defined graded association exists between severity of reduction in baseline eGFR and progressively higher risk of AKI [24]. Furthermore, it should be noted that 22 of the total 61 patients underwent vascular surgery, mainly open abdominal aneurysm repair, a procedure known to be associated with a postoperative AKI incidence of up to 26% depending on the AKI definition [25].

It is noteworthy that in the AKI group, a significant decline in eGFR with a concomitant rise in serum creatinine and cystatin C was already evident at the recovery room, immediately following surgery. This finding is in line with previous observations in cardiac surgery patients [26], as well as surgical ICU patients [27], where an immediate postoperative elevation in serum creatinine and cystatin C has also been described. However, one should keep in mind that in the early stages of AKI, the diagnostic value of serum creatinine is limited for several well-known reasons [28]. More importantly, the various eGFR equations that have been developed are intended for diagnosing and staging CKD patients in steady-state conditions rather than estimating creatinine clearance during an AKI episode [29].

The most interesting finding of the present study was the significant association between an abnormal preoperative UACR and the development of postoperative AKI, regardless of preoperative renal function or other comorbidities. In fact, the presence of a UACR >30 mg·gr^−1^ resulted in a five-fold higher risk of AKI development. In recent years, albuminuria has been identified as a significant risk factor for AKI and adverse long-term outcomes, both in the general population [30,31] and in cardiac surgery patients [32,33]. Our findings are in line with the results of a large prospective study in the general population (the Atherosclerosis Risk in Communities (ARIC) study) [24], showing a significant risk of hospitalizations and/or death from AKI in people with increased baseline UACR. Preoperative proteinuria, independent of preoperative eGFR, was not only associated with the risk of AKI, but was also a powerful independent risk factor for long-term all-cause mortality and end stage renal disease (ESRD) after cardiac surgery [32,33]. However, to the best of our knowledge, this is the first study reporting a strong relationship between pre-operative albuminuria and postoperative AKI after non-cardiac surgery, a factor that has been overlooked in existing predictive models.

CKD is the most consistent pre-existing condition associated with a high risk of AKI in almost every relevant study. While the definition of CKD includes many parameters, with the most prevalent being albuminuria, this parameter has been largely neglected in most studies where data on urine albumin were generally lacking [12]. Albuminuria represents a state of generalized endothelial dysfunction and is recognized as one of the most important risk factors for cardiovascular and renal events [34,35]. It is not surprising that baseline UACR was identified as an independent prognostic factor for postoperative AKI for two reasons: First, the recognition of increased UACR reclassifies patients with normal eGFR to stage I CKD, which increases the risk for AKI; and second, it unmasks an already existing renal pathology that is not detected by serum creatinine alone. In this context, angiotensin converting enzyme inhibitors (ACEi(s)), an established anti-proteinuric therapy, have been shown to reduce the incidence of postoperative AKI and all-cause mortality by 17% and 9%, respectively [36]. Thus, we can hypothesize that the protective effect of ACEi(s) may be mediated not only via the intra-renal hemodynamic effects, but also due to their anti-proteinuric properties. Nevertheless, since this is an observational study, we can only report associations and cannot claim causality.

Blood loss and the need for intraoperative transfusion of red blood cells and fresh frozen plasma were found to be associated with the development of postoperative AKI. These factors represent well-known risks for AKI [37]. A meta-analysis of 20 randomized controlled trials on perioperative hemodynamic optimization identified several interventions associated with significant reduction in the incidence of postoperative AKI [38], including optimization of intravascular volume, cardiac output, and oxygen delivery, emphasizing the importance of intraoperative preservation of renal perfusion. Furthermore, multiple studies have found an association between RBC transfusion and renal dysfunction in patients undergoing cardiac and vascular surgery. It is known that stored RBCs undergo changes known as “storage lesion”, i.e., irreversible changes in RBC deformity causing stronger adherence to vascular endothelium with a resulting decreased microvascular flow [38,39].

Regarding preservation of renal perfusion, intraoperative blood pressure management is regarded as a key element. Recent observational studies identified episodes of intraoperative hypotension as an independent risk factor for AKI development [40,41]. This observation has been further strengthened by a multi-center randomized controlled trial [42], where an intraoperative strategy targeting an individualized systolic blood pressure (within 10% of patient baseline values) achieved an absolute AKI risk reduction of 16% compared to standard management, emphasizing the importance of individualized patient care. We were not able to reproduce these findings in our study population, where according to anesthetic charts, time-averaged mean arterial pressure was higher than 65 mmHg and did not differ between the two groups. However, a larger proportion of patients in the AKI group required intraoperative vasopressor support, which might be regarded as an indirect indicator of severe intraoperative hypotensive episodes.

Another intraoperative factor significantly and independently associated with development of postoperative AKI was the intraoperative infusion of a high chloride load. This finding is in line with experimental, as well as clinical data, showing that chloride infusion-induced hyperchloremia causes renal vasoconstriction resulting in cortical hypoperfusion [43,44]. A growing body of evidence from observational studies suggests that volume loading with chloride-rich solutions is associated with kidney dysfunction, as compared with the use of balanced solutions [45,46,47]. A recent meta-analysis including more than 6000 patients concluded that the perioperative use of chloride-rich crystalloids, as compared to the use of balanced solutions, increased the risk of AKI [48].

## 5. Conclusions

In conclusion, the present study showed that in patients developing postoperative AKI, a reduction of the eGFR can be detected very early in the postoperative period. An intraoperative chloride load higher than 500 mEq, as well as a pre-operative urine albumin to creatinine ratio >30 mg·g^−1^, were significantly and independently associated with postoperative AKI development. However, since the study patients had several comorbidities and some degree of pre-existing renal dysfunction rendering them more susceptible to future renal injury, the implications of these results might be limited to patients with similar comorbidities and not be applicable to otherwise healthy surgical patients. Nevertheless, the presence of preoperative albuminuria seems to be a significant, yet neglected, independent risk factor for postoperative AKI. Bearing in mind that the strength of association and degree of external validity of this risk association needs further evaluation. We suggest that albuminuria should be included in the routine preoperative patient evaluation for major abdominal surgery and considered as a possible prognostic factor in future AKI risk prediction models.

## Figures and Tables

**Table 1:** AKI * diagnosis based on AKIN ** creatinine criteria. (Patients receiving RRT *** are considered to have met criteria for Stage 3, irrespective of the stage they were at the commencement of RRT).

AKI Staging	Definition
Stage 1	1.5-fold increase in sCr or increase of ≥0.3 mg·dL^−1^
Stage 2	2-fold increase in sCr
Stage 3	3-fold increase in sCr or sCr ≥ 4 mg·dL^−1^, with acute rise of ≥0.5 mg·dL^−1^

* AKI = acute kidney injury; ** AKIN = Acute Kidney Injury Network; sCr = serum creatinine; *** RRT = renal replacement therapy.

**Table 2:** Baseline clinical and demographic characteristics according to AKI status.

	Non-AKI Group (*n* = 50)	AKI Group (*n* = 11)	*p* Value	Odd’s Ratio (95% CI)
**Demographics**				
Mean age, (years ± SD)	66.9 ± 10	68.2 ± 10.4	0.699	1.01 (0.95–1.08)
Male gender, (*n*)	33	8	0.67	1.02 (0.92–1.13)
Mean BMI, kg·m^−^²	27.2 ± 4.2	27.7 ± 5.6	0.721	1.03 (0.89–1.19)
**ASA Classification, (*n*)**				
ASA II	27	4	0.087	1.0
ASA III	20	6	0.076	3.9 (0.73–33.80)
ASA IV	3	1	0.249	4.8 (0.33–70.40)
**Comorbidities, (*n*)**				
Hypertension	31	6	0.738	1.4 (0.20–2.75)
CKD	34	9	0.481	2.1 (0.41–10.95)
Stage I	4	2	0.226	4 (0.42–37.78)
Stage II	25	5	0.600	1.6 (0.28–9.26)
Stage III	5	2	0.301	3.2 (0.35–28.94)
CAD	14	4	0.717	1.5 (0.37–5.81)
CHF	11	5	0.067	3.8 (0.98–14.70)
DM	14	5	0.137	3 (0.76–11.54)
COPD	19	6	0.174	3.1 (0.80–12.09)
**Surgical Procedures, (*n*)**				
Other abdominal Surgery	35 (89.7%)	4 (10.3%)	0.079	
Vascular surgery	15 (68.2%)	7 (31.8%)	0.035	4.1 (1.04–16.06)

Mean (SD): mean and standard deviation (in brackets), *n*: number of patients OR (95% CI): Odd’s ratio with 95% confidence intervals (in brackets), *p*-values from Pearson’s chi-square or Fisher’s exact test. Abbreviations: AKI: Acute Kidney Injury; BMI: Body Mass Index; CKD: Chronic Kidney Disease; CAD: Coronary Artery Disease; CHF: Congestive Heart Failure; DM: Diabetes Mellitus; COPD: Chronic Obstructive Pulmonary Disease.

**Table 3:** Baseline laboratory values according to AKI status (expressed as mean ± SD, except UACR which is expressed in numbers of patients).

	Non-AKI Group (*n* = 50)	AKI Group (*n* = 11)	*p* Value	Odd’s Ratio (95% CI)
Serum Creatinine, mg·dL^−1^	0.87 (±0.23)	1.02 (±0.21)	0.063	12.6 (0.80–198.7)
Serum Cystatin C, mg·L^−1^	0.76 (±0.19)	0.82 (±0.28)	0.319	5.20 (0.21–126.7)
eGFR, mL·min^−1^ 1.73 m^−2^	83.92 (±15.15)	75.27 (±15.97)	0.095	0.97 (0.93–1.01)
Urine Albumin, mg·L^−1^	24.1 (±50.7)	325 (±729.9)	0.042	1.01 (1.00–1.01)
Urine Creatinine, g·L^−1^	1.1 (±0.6)	1.2 (±0.7)	0.344	1.66 (0.58–4.66)
UACR > 30 mg·gr^−1^, (*n*)	9 (18.0)	6 (54.5)	0.011	5.47 (1.36–21.92)
FeNa, %	0.8 (±0.7)	0.8 (±0.8)	0.919	1.05 (0.40–2.75)
FeUrea, %	41.4 (±12.7)	37.2 (±10.5)	0.303	0.97 (0.92–1.03)

Mean (SD): mean and standard deviation (in brackets), *n*: number of patients. OR (95% CI): Odd’s ratio with 95% confidence intervals (in brackets), *p*-values from Pearson’s chi-square or Fisher’s exact test. Abbreviations: AKI: Acute Kidney Injury; eGFR: estimated Glomerular Filtration Rate; UACR: Urine Albumin to Creatinine Ratio; FeNa: Fractional Excretion of Sodium; FeUrea: Fractional Excretion of Urea.

**Table 4:** Intraoperative variables according to AKI status (expressed as mean ± SD, except Vasopressors which are expressed in numbers of patients).

Non-AKI Group (*n* = 50)		AKI Group (*n* = 11)	*p* Value	Odd’s Ratio (95% CI)
**Intraoperative Variables** **(mean** **±** **SD)**				
Urine output (mL·kg^−1^·hr^−1^)	2.1 (±2.0)	1.1 (±0.8)	0.151	0.37 (0.13–1.1)
Mean Arterial Pressure (mmHg)	78.7 (±6.9)	79.6 (±10.5)	0.734	1.01 (0.93–1.10)
Vasopressor administration, (*n*)	32 (64.0)	8 (72.7)	0.581	1.67 (0.86–2.65)
Blood loss (mL)	479 (±508.5)	1345.5 (±1336.7)	0.002	1.06 (1.02–1.1)
**Intraoperative Fluids** **(mean ± SD)**				
Total crystalloids (mL)	2441 (±1195)	3018 (±1497)	0.171	1.00 (1.00–1.00)
Total colloids (mL)	865 (±354)	1046 (±611)	0.191	1.00 (1.00–1.00)
RBC transfusion (units)	1.1 (±1.4)	2.3 (±2.4)	0.042	1.49 (1.03–2.01)
FFP transfusion (units)	1.2 (±1.4)	2.6 (±2.1)	0.006	1.69 (1.11–2.56)
PLT transfusion (units)	0.04 (±0.28)	0.4 (±1.2)	0.088	2.04 (0.73–5.71)
Chloride load (mEq)	458.6 (±179.8)	583.9 (±191.5)	0.043	1.03 (1.01–1.05)

Mean (SD): mean and standard deviation (in brackets), *n*: number of patients; OR (95%CI): Odd’s ratio with 95% confidence intervals (in brackets); *p*-values from Pearson’s chi-square or Fisher’s exact test. AKI: Acute Kidney Injury; RBC: Red Blood Cells; FFP: Fresh Frozen Plasma; PLT: Platelets.
